# The response of soil microbial community to application of organic amendment to saline land

**DOI:** 10.3389/fmicb.2024.1481156

**Published:** 2025-01-06

**Authors:** Peifei Cong, Pengfei Huang, Zhisheng Huang

**Affiliations:** ^1^Institute of Farmland Irrigation, Chinese Academy of Agricultural Sciences, Xinxiang, China; ^2^DeepBlue Academy of Sciences, Shanghai, China; ^3^Yucheng Comprehensive Experiment Station, Key Laboratory of Ecosystem Network Observation and Modeling, Institute of Geographic Sciences and Natural Resources Research, Chinese Academy of Sciences, Beijing, China; ^4^Clinical Research Center for Mental Disorders, Shanghai Pudong New Area Mental Health Center, Tongji University School of Medicine, Shanghai, China; ^5^Knowledge Representation and Reasoning (KR&R) Group, Vrije Universiteit Amsterdam, Amsterdam, Netherlands; ^6^Haoxinqing Health Industry Group, Beijing, China

**Keywords:** functional diversity, salinity increase, organic remediation, community level physiological profile, microbial community

## Abstract

**Introduction:**

The salinization of coastal soils is a primary cause of global land degradation. The aim of this study was to evaluate the effect of organic amendment on the soil microbial community within a saline gradient.

**Methods:**

The study was designed with five levels of electrical conductivity (EC): 0.33, 0.62, 1.13, 1.45 and 2.04 ds m^−1^. By conducting indoor potted plant experiments, determine the effects of applying microbial organic fertilizer on the physicochemical properties of soil and the structure of soil microbial communities under different salinity concentrations.

**Results:**

Compared with the control, higher OM content, total N, and higher crop biomass were observed in samples with organic amendment at the same salinity level. At the same salinity levels, the mean bacterial activity (AUC) and the mean number of substrates were higher than in the soil without organic amendment according to analyses by means of Biolog ECO MicroPlate. The results of canonical correspondence analysis indicate that after the application of organic amendments, the composition of loam and clay replaces soil pH, and aboveground biomass replaces root biomass as key indicators closely monitoring Community level physiological profiling (CLPP). In soil with the same salinity level, the application of microbial organic fertilizer led to an increase in the proportion of Actinobacteriota and a decrease in the proportion of Chloroflexi. In 0.3dS m-1 soil, the abundance of actinomycetes increased from 23% to 27% after application of microbial organic fertilizer, while the abundance of basidiomycetes decreased from 20% to 6%. In addition, after the application of microbial organic fertilizer, RB41, blastococcus and solirubrobacter significantly increased, while Melothermus and Herpetosiphon significantly decreased.

**Discussion:**

This study provides a strong theoretical basis for using microbial organic fertilizers to improve saline-alkali soil.

## 1 Introduction

The salinization of coastal soils is a primary cause of global land degradation. Soil salinity in coastal areas is the main cause for the global land degradation that has been targeted for remediation. Many studies have revealed that application of organic amendment is a good way to improve saline-alkali lands. López-Valdez et al. (2010) demonstrated that addition of microorganisms with sludge increases NH_4_^+^ immobilization and induces fixation of NO_3_^−^ in an alkaline saline soil. It was found that organic matter (OM) can stimulate the growth of arbuscular mycorrhizal fungi, which can enhance plant growth and health on saline dryland (Hammer et al., 2011; Songlin et al., 2024). OM can increase available water content (Temesgen et al., 2023) and improve the soil structure, which is important for the formation of macro-aggregates (Rackley, 2023). Klopp et al. found that application of microbes with gypsum can noticeably increase saturated hydraulic conductivity of saline (Klopp and Daigh, 2020). Application of organic amendment to a saline alkali soil was found to improve the soil nutrient content and the abundance of soil organisms (Hou et al., 2021). Nevertheless, the precise effects of different kinds of organic amendments on nutrient mineralization and immobilization depends on the chemical composition and the potential of the soil microbial community (Landry Rossdeutsch et al., 2020). Most of recent studies have been conducted to research the effect of organic amendment from a certain salt concentration range but have hardly ever evaluated this effect across the soil salinity gradient.

The most important processes in a soil are inseparable from the microbes in the soil. These microbes can enhance the availability of nutrients. Arbuscular mycorrhizal fungi can grow into the smallest soil micropores and help plants absorb water and nutrients (Somak et al., 2022). Some of the soil microbes play an important role in making nutrients available to plants and microbes by recycling them in the soil (Adnane et al., 2018). The biochemical substances produced by soil microbes can improve the formation of the soil aggregate structure. In a saline soil, the biota is adversely affected because salinity alters the osmotic and matric potential of the soil solution. In recent years, biological methods were used as a practical way to alleviate various types of soil stress. For example, plant growth-promoting rhizobacteria (PGPR) can alleviate the adverse effects of stress on plant growth (Adnane et al., 2018). Daei reported the species of arbuscular mycorrhizal fungi that were isolated from soils under salinity conditions. The right combination of fertilizers and soil microbes enhances the biofertilizer efficiency (Adesemoye et al., 2008). Nevertheless, Elgharably and Marschner (2011) showed that salinity depresses microbial biomass. Most studies have shown that soil respiration decreases with the increasing electrical conductivity (EC) of the soil (Setia et al., 2010).

It has been reported that the intensive application of organic amendment increased soil microbial biomass and activity and created distinct microbial community compositions (Hasbullah and Marschner, 2015). The soil in saline-alkali lands is detrimental to plant growth, and its properties differ from those of regular soil. Therefore, it is necessary to study the effects of soil amendments on saline-alkali soil improvement. Plant roots can produce organic compounds including carbohydrates, organic and carboxylic acids. Those products can serve as food for soil microbes and affect microbial activities (Benizri and Amiaud, 2005). Microbial characteristics also affect vegetation such as biomass (Gömöryová et al., 2013). In addition, fertilization has an impact on the microbial community structure, and then physiochemical properties of the soil change under the influence of the changes of the soil microbial functions. On the other hand, it is unknown which biotic of abiotic factors are associated with soil salinity by organic amendment application. The relation between soil characteristics and bacterial functional diversity after application of organic amendment is also not clear.

Soil microbial functional diversity is widely used to evaluate soil processes and ecological functions (Xiang et al., 2023). Biolog ECO MicroPlate were found to be an appropriate analytical method for discrimination between communities by selecting carbon substrates (Gabriela et al., 2023). Using Biolog ECO MicroPlate, Klimek determined the relation between the diversity of a vascular-plant community and overall microbial activity in the soil (Klimek et al., 2015). Jin reported that average well color development (AWCD) decreases and C utilization patters change with the increasing soil salt concentration (Jin et al., 2015). CLPP based on Biolog ECO MicroPlate offers common methods for research on functional diversity of soil bacteria (Klimek et al., 2016).

To close the knowledge gap, we carried out a pot assay. The diameter of the pot is 35 centimeters and the diameter of the pot is 35 centimeters. The objectives of the study were: (1) to evaluate the effect of organic amendment on soil physiochemical characteristics and ecological characteristic of soil microorganisms through CLPP in the salinity gradient; (2) to explore the relation between an array of microbiological and soil characteristics and plant biomass after organic amendment applied in saline soils.

## 2 Materials and methods

### 2.1 Field sampling and characteristics

Five soils with similar texture (loam) were collected from the Wudi County of Shandong Province, China (37.73 N, 117.6 E), on April 22, 2021 at 0 to 20 cm depth. This area is affected by the North China plain, is low and flat, and is located in a warm temperate semi-humid continental monsoon climatic zone. Seawater encroachment and irrigation with saline water are the causes of soil salinity. The EC was 0.30, 0.62, 1.13, 1.45, 2.04 ds m^−1^ in the five soils. This range was chosen on the basis of other studies to induce an increase from moderate to strong respiration by means of salinity (Chowdhury et al., 2011; Rui et al., 2021). Selected soil properties are shown in Table 1. The soils were pre-incubated for 10 days at 50% of water holding capacity (WHC) to stabilize their activity. The samples were for biological assays stored at 4°C. They were performed for the CLPP. The samples were for DNA extraction stored at −80°C.

**Table 1:** Soil characteristics.

parameter	EC_1:5_ (ds m^−1^)				
	0.33	0.62	1.13	1.45	2.04
EC_e_ (ds m^−1^)	10.6 ± 0.6	13.8 ± 3.8	19.6 ± 3.6	23.2 ± 0.26	29.9 ± 5.9
Sand (%)	43	49	47	47	45
Silt (%)	37	34	35	36	35
Clay (%)	20	17	18	17	20
Water holding capacity (%)	29	30	32	33	35
pH	8.84 ± 0.06	8.80 ± 0.05	8.94 ± 0.06	8.97 ± 0.07	9.00 ± 0.04
Total organic carbon (%)	0.8 ± 0.03	0.7 ± 0.03	0.6 ± 0.04	0.7 ± 0.02	0.6 ± 0.06
Total *N* (g kg^−1^)	0.99 ± 0.06	0.85 ± 0.07	0.79 ± 0.08	0.73 ± 006	0.57 ± 0.07
Available nutrients					
P (mg kg^−1^)	12 ± 5.3	11.58 ± 5.7	11.29 ± 6.2	10.38 ± 6.4	9.18 ± 7.2
K (mg kg^−1^)	115 ± 21	133 ± 32	135 ± 24	143 ± 19	156 ± 17
NH_4_-N (mg kg^−1^)	5.5 ± 0.91	5.3 ± 0.88	4.8 ± 0.92	4.5 ± 0.99	4.0 ± 0.82
NO_3_-N (mg kg^−1^)	5.5 ± 0.09	5.2 ± 009	4.4 ± 0.08	3.2 ± 0.07	2.6 ± 0.43
Soluble cations (1:5 soil: water extract)					
Na^+^ (mmol L)	0.41 ± 0.009	3.32 ± 0.12	9.61 ± 0.42	12.98 ± 0.22	15.54 ± 0.32
Ca^+^(mmol L)	0.52 ± 0.009	0.41 ± 0.023	0.65 ± 0.028	0.78 ± 0.021	0.89 ± 0.009
Mg^+^ (mmol L)	0.23 ± 0.009	0.26 ± 0.012	0.22 ± 0.019	0.76 ± 0.023	0.81 ± 0.021
SAR_1:5_	0.6	5.7	14.6	14.8	16.9

### 2.2 Materials

The organic amendment was fermented and refined for more than 60 days. The principal materials were animal excrement and plant straw. The straw was machine ground to - 5 mm lengths before organic composting. The materials were mixed with fermentation liquor in a ratio of 1: 200. The animal excrement and plant straw were obtained from the experimental station. The fermentation liquor was provided by the ETS biological science and technology development company (Tianjin). It includes more than 60 strains of microbes. The organic amendment was analyzed and showed: EC = 0.3 ds m^−1^; pH = 5.8; total *N* = 17.8 g kg^−1^; total *C* = 13.25%; C/N ratio = 7.43; available *p* = 12 g kg^−1^ and available *K* = 18.02 g kg^−1^. Chemical fertilizer was obtained from experimental station, which N + P_2_O_5_ + K_2_O was 15–15-15, and total Nutrient was ≥45%.

### 2.3 Experiment design

The pot experiment was carried out in the Yucheng agricultural ecological experimental station greenhouse. The soils were the same as those in the incubation experiment and corresponded to five levels of EC: 0.30, 0.62, 1.13, 1.45, and 2.04 ds m^−1^. Eight kilograms (wet weight) of soil was put in each pots. The size of the ceramic pots was 22 × 19 × 25 cm. There were 10 treatments in the pot experiment. Before filling the pots with soil, we stirred the fertilizer into soil: 64 g of organic amendment (300 [kg N] ha^−1^) was added in the organic amendment group of soil samples. Treatment without organic fertilizer was used as a control in the experiment. In order to balance the total nutrients (N + P_2_O_5_ + K_2_O), 8 g of chemical fertilizer was added in the control group. There were 10 treatments in the pot experiment: the EC value of soil was 0.33 ds m^−1^ with organic amendment, 0.33 ds m^−1^ with chemical fertilizer; the EC value of soil was 0.62 ds m^−1^ with organic amendment, 0.62 ds m^−1^ with chemical fertilizer, the EC value of soil was 1.13 ds m^−1^ with organic amendment, 1.13 ds m^−1^ with chemical fertilizer, the EC value of soil was 1.45 ds m^−1^ with organic amendment, 1.45 ds m^−1^ with chemical fertilizer, the EC value of soil was 2.04 ds m^−1^ with organic amendment, 2.04 ds m^−1^ with chemical fertilizer. Each treatment was performed in triplicate.

We watered each pot with the same amount of water using a sprinkler to keep the soil moist but not water-logged. The crop studied was spring maize (*Zea mays*) variety Zhengdan 958, sown on April 29, 2021, and harvested on October 8 of that year.

### 2.4 Sampling and analytical methods

Soil from each pot was sampled in the September 2021. One sample was collected with a 3-cm-diameter soil auger. Three samples were collected from four corners and obtain one mixed soil sample per plot. The fresh soil was sampled for analysis of the microbial community. Subsamples were air-dried and used for measurements of soil SOC, total N, pH. After 5 months of cultivation, we weighted the fresh and dried plants.

Soil pH were determined with standard procedures. The EC was measured in a 1:5 (soil: water, w/w) ratio mixture after 1 h of end-over-end shaking. The EC of a saturated pasted (EC_e_) was calculate by the equation: EC_e_ = (14.0–0.13 × clay %) × EC_1:5_ (Yan and Marschner, 2012). Exchangeable NH_4_^+^ and NO_3_^−^ concentrations were determined with a continuous-flow analyzer. Soil total N was analyzed using the Kjeldahl digestion method. Organic C concentrations was determined by the wet oxidation-redox titration method (Adesemoye et al., 2008). Soil bulk density was measured by the clod method (Blake 1965). WHC was measured by placing thoroughly wetted soils in a sintered glass collected to a 100 cm water column, allowing them to drain for 48 h (Yan and Marschner, 2012).

### 2.5 Biolog ECO MicroPlate analysis

Biolog ECO MicroPlate (Biolog, Harward, CA, USA) was used to determine the nutritional versatility of microbial metabolic potential from the various soil treatments (Klimek et al., 2016; Xuekai et al., 2021). There were 96 wells in the Biolog Eco MicroPlates containing 31 carbon (C) sources and one without a C source and tetrazolium dye as the substrate utilization indicator.^1^ There were three parallel repeats for each C source (Supplementary Table S1). The C sources were classified into six substrate guilds: amino acids, carbohydrates, carboxylic acids, miscellaneous, amines, and polymers (Encarnación et al., 2021). In brief, 10 g of fresh soil was added to 90 mL of a NaCl solution (0.85%) in a 100 mL triangle flask. Soil solutions in the triangle flask were shaken for 30 min at 220 rpm. Next, they were left to stand for 15 min, and then we collected supernatants. Ten-fold dilutions (10^−1^ and 10^−3^ dilutions) in a sterile NaCl solution (0.85%) were prepared. The final diluent was injected into Biolog Microtiter plates with an eight channel sampler (150 μL per well). We put the plates into a biochemical incubator at 25°C. When C source was utilized by microorganisms, the color would change in the wells. The absorbance measured at 590 nm every 24 h and recorded with Biolog software (Biolog, Hayward, USA).

Area under the curve (AUC) served as a common indicator of bacterial activity (Ju and Zhang, 2015). The formula as follows:


$$AUC=\overset{N}{\underset{i=1}{\sum }}\overset{n-1}{\underset{t=1}{\sum }}\frac{A_{n}+A_{n+1}}{2}\times \left(t_{n+1}-t_{n}\right)$$

where A_n_ and A_n + 1_ are the absorbance of each single well at times points t_n_ and t_n + 1,_ *n* means the day of measurement, and *N* means the number of substrates in the plate (Koner et al., 2021). The comparison of CLPP for different samples was conducted at the same AWCD, which was 0.09 (Gabriela et al., 2023).

### 2.6 Soil aggregation

Clods from moist samples were gently broken apart. One part of the soil was used to determine the size distribution of aggregates using the method of wet sieving (Yu et al., 2022). Briefly, there was a series of three sieves (2000-, 250-, and 53-μm pore size). A 60 g subsample of a soil was passed through the 2000-μm sieve, and submerged in deionized water for 5 min at room temperature. To separate the aggregates, we moved the sieve 3 cm up and down, 50 times for 2 min. Next, the fraction retained on the 2000-μm sieve was backwashed into an aluminum box. The 250- to 2000-μm aggregates were collected by the means of the 2000-μm mesh. The <250 μm aggregates were collected by the 250-μm sieve, and silt and clay were collected after passing through the 53-μm sieve. The aggregates were oven dried (at 40°C).

### 2.7 DNA extraction and high-throughput qPCR

After genomic DNA extraction, the extracted genomic DNA was detected by 1% agarose gel electrophoresis. Specific primers with barcode were synthesized according to the specified sequencing region. In order to ensure the accuracy and reliability of subsequent data analysis, two conditions should be met: (1) Low cycle number amplification should be used as much as possible; (2) Ensure that the number of cycles amplified for each sample is consistent. Random selection of representative samples for pre-experiment to ensure that in the minimum number of cycles, the majority of samples can be amplified with appropriate concentration of products. PCR was performed using TransGen AP221-02: TransStart Fastpfu DNA Polymerase. PCR instrument: ABI GeneAmp&reg;Type 9,700;

All samples were carried out according to the formal experimental conditions, with 3 replicates per sample. PCR products of the same sample were mixed and detected by 2% agarose gel electrophoresis. The PCR products were recovered by using AxyPrepDNA gel recovery kit (AXYGEN) and elution by Tris_HCl 2% agarose electrophoresis detection.

### 2.8 Analysis of microbial communities

To optimize the extraction of repeat sequences, easy to reduce redundant computation analysis among process, get rid of the single sequence with no duplication,^2^ in accordance with the 97% similarity to a repetitive sequence (not including single sequence) OTU clustering, the representative sequence of OTU is obtained by removing chimera in the process of clustering. Map all optimized sequences to OTU representative sequences, select sequences that are more than 97% similar to OTU representative sequences, and generate OTU table. In order to obtain the species classification information corresponding to each OTU, 97% of the OTU representative sequences with similar levels were analyzed by using RDP classifier Bayesian algorithm.

Software and algorithm: Qiime platform, RDP, confidence threshold is 0.7.

### 2.9 Statistical analyses

Two-way ANOVA was performed to evaluate the differences in the mean physicochemical properties of a soil by means of the SPSS 21 statistical software. The significance level was <0.05. The significant differences were analyzed by Tukey’s test.

The significant differences in CLPP under the influence of salinity and fertilizer were subjected to analysis of similarities (ANOSIM). It yields *p* value and *R* value. The *R* values were used to assess the degree of correlation. *R* ranged from 0 to 1. *R* = 1 means that the correlation between factors was very high. *R* = 0 means that the null hypothesis is correct (no correlation between samples). The global *R* value represented overall dissimilarity among the groups of samples.

We identified the contribution of chemical guilds to the similarity percentage (SIMPER) (Hannah et al., 2021). The average Bray–Curtis dissimilarity was used to evaluate differences between the factors. In addition, the standard deviation denotes the consistency of how a given variable contributes to the dissimilarity between soil salinities or fertilizers.

The relation between the CLPP and soil characteristics at different soil salinity levels and fertilizer treatments was measured with canonical correspondence analysis (CCA). The latter is a multivariate constrained ordination method. It can find the main gradients among factors.

## 3 Results

### 3.1 Soil physical structure properties

The bulk density was affected by the interaction of salinity and fertilizer. For samples without organic fertilizer, the bulk density in soils with EC ≥ 1.13 dS m^−1^ was higher than in soils with EC ≤ 1.45 dS m^−1^. With the increasing salinity, the proportion of macro-aggregates (> 250 μm) decreased, whereas the silt + clay fraction (< 53 μm) increased. When soil EC values were 0.33, 0.62, and 1.13 ds m^−1^, organic amendment applications significantly increased the proportion of >250 μm aggregates by 20.03, 18.51, and 20.83%, respectively, and reduced the proportions of <53 μm aggregates by 9.95, 16.90, and 17.00%, compared to the control (Supplementary Table S2). Therefore, application of organic amendment significantly increased the proportion of macro-aggregates and lowered the silt + clay fraction content (*p* < 0.05). The differences in proportions of aggregates in soils with EC values of 1.45 and 2.04 ds m^−1^ were not significantly different compared with their controls. Salinity significantly affected the soil aggregate structure. The proportions of aggregates at all levels except the micro-aggregates (250–2000 μm) were influenced remarkably by the application of the organic amendment. Nevertheless, highly significant interactions between organic amendment and salinity were found in terms of the aggregate structure characteristics (Table 2).

**Table 2:** Mean values and standard deviations for physical structure properties of the soils.

Soil salinity (ds m^−1^)	Soil physical structure parameter				
	Bulk density (g cm^−3^)	Macro-aggregate (>2,000 μm)	Macro-aggregate (250–2,000 μm)	Micro-aggregate (53–250 μm)	Silt + clay fraction (<53 μm)
No applying organic amendment					
0.30	1.37 (0.01)bA	13.74 (0.44)aB	25.05 (0.69)aB	29.35 (0.21)aA	31.86 (1.01)eA
0.62	1.39 (0.04)bB	13.36 (0.12)bB	16.51 (1.22)bB	31.26 (0.63)bA	38.87 (1.71)dA
1.13	1.39 (0.02)bA	9.77 (0.24)cB	11.31 (0.59)cA	30.03 (0.50)cB	48.89 (0.54)cA
1.45	1.47 (0.00)aA	9.15 (0.54)dA	8.12 (0.23)dAB	27.72 (0.33)dB	55 (1.52)bA
2.04	1.44 (0.01)aA	3.50 (0.13)eB	5.00 (0.69)eAB	24.67 (0.38)eA	66.82 (1.64)aA
Applying organic amendment					
0.30	1.37 (0.06)bA	14.33 (0.98)bA	32.23 (1.36)aA	18.41 (2.72)cB	35.03 (2.44)cA
0.62	1.46 (0.02)aA	15.66 (0.89)aA	19.74 (0.35)bA	32.29 (1.00)aA	32.3 (0.41)cB
1.13	1.43 (0.01)abA	11.66 (0.43)cA	13.81 (1.11)cA	33.95 (1.15)aA	40.58 (0.71)cB
1.45	1.43 (0.01)abB	9.81 (0.02)dA	9.82 (0.84)dA	29.86 (0.82)bA	55.88 (4.16)bA
2.04	1.42 (0.02)abA	4.05 (0.09)eA	6.33 (0.67)eA	26.24 (1.98)bA	63.38 (3.27)aA
Salinity	0.007	0.000	0.000	0.038	0.000
Fertilizer	0.322	0.026	0.002	0.449	0.012
Interaction	0.000	0.000	0.000	0.000	0.000

Values are means (*n* = 3) with standard error; significant differences between salinities were indicated by small letters (a, b, c); significant differences between fertilizers were indicated by capital letters (A, B).

### 3.2 Chemical characteristics and crop biomass

EC was higher than the initial values before the experiment, because the irrigation water contains some salt. At the same salinity level, we found that in the samples with organic amendment, EC increased less than it did in the control samples. Except for EC 0.3 ds m^−1^, the control pH values were lower than those in the samples with organic amendment. This is because controls were supplemented with chemical fertilizer, which contains alkaline substances. Compared with the control, higher OM content, total N, and higher crop biomass were observed in samples with organic amendment at the same salinity level; organic amendment application increased the OM content and total N by 10.69–28.72% and 33.33–76.92%, respectively. In addition, the increase was smaller in the higher salinity soils (> 1.13 ds m^−1^). EC, pH, OM, total N, and biomass were affected significantly by salinity, organic amendment status, and the interaction of thereof (Table 3).

**Table 3:** Mean values and standard deviations for chemical characteristics and plant biomass.

Soil salinity (ds m^−1^)	Soil chemical and biomass parameter Crop biomass					
	EC (ds m^−1^)	pH	OM (g kg^−1^)	Total *N*	Root biomass	Shoot biomass
No applying organic amendment						
0.30	0.43eA (0.01)	8.01cA (0.08)	6.72aB (0.11)	0.82aB (0.09)	17.42aA (0.78)	175.99abAB (11.59)
0.62	0.96dA (0.02)	8.11bcA (0.06)	5.76bB (0.05)	0.65bB (0.07)	14.76bB (4.89)	179.63aA (30.08)
1.13	1.59cA (0.01)	8.19bB (0.02)	4.43cB (0.10)	0.57bA (0.04)	14.54bB (3.32)	192.62aB (20.36)
1.45	1.73bA (0.04)	8.03bcA (0.05)	3.93dB (0.09)	0.41cA (0.09)	12.91cAB (1.73)	133.89cB (9.39)
2.04	2.50aA (0.04)	8.48aA (0.02)	3.24eB (0.05)	0.38cB (0.03)	9.34dA (0.05)	159.66bA (4.17)
Applying organic amendment						
0.30	0.41eA (0.01)	7.99cA (0.04)	8.65aA (0.14)	1.32aA (0.10)	21.62aA (.62)	219.92aA (2.69)
0.62	0.78dB (0.01)	8.29bA (0.14)	7.01bA (0.11)	1.15bA (0.06)	19.56bA (3.84)	214.36aA (14.32)
1.13	1.34cB (0.04)	8.37bA (0.02)	5.64cA (0.10)	0.76cA (0.12)	19.58bA (0.61)	219.38aA (12.58)
1.45	1.53bA (0.05)	8.21bcA (0.06)	4.35dA (0.14)	0.62cdA (0.03)	15.1cA (5.12)	162.00bA (8.20)
2.04	2.16aB (0.04)	8.67aA (0.18)	3.67eA (0.10)	0.50dA (0.06)	9.79dA (1.16)	153.10bA (11.05)
Salinity	0.000	0.000	0.000	0.000	0.000	0.000
Fertilizer	0.000	0.001	0.000	0.000	0.000	0.049
Interaction	0.000	0.000	0.000	0.000	0.000	0.000

Values are means (*n* = 3) with standard error; significant differences between salinities were indicated by small letters (a, b, c, d, e); significant differences between fertilizers were indicated by capital letters (A, B).

### 3.3 Characteristics of soil bacteria

**Figure 1:**
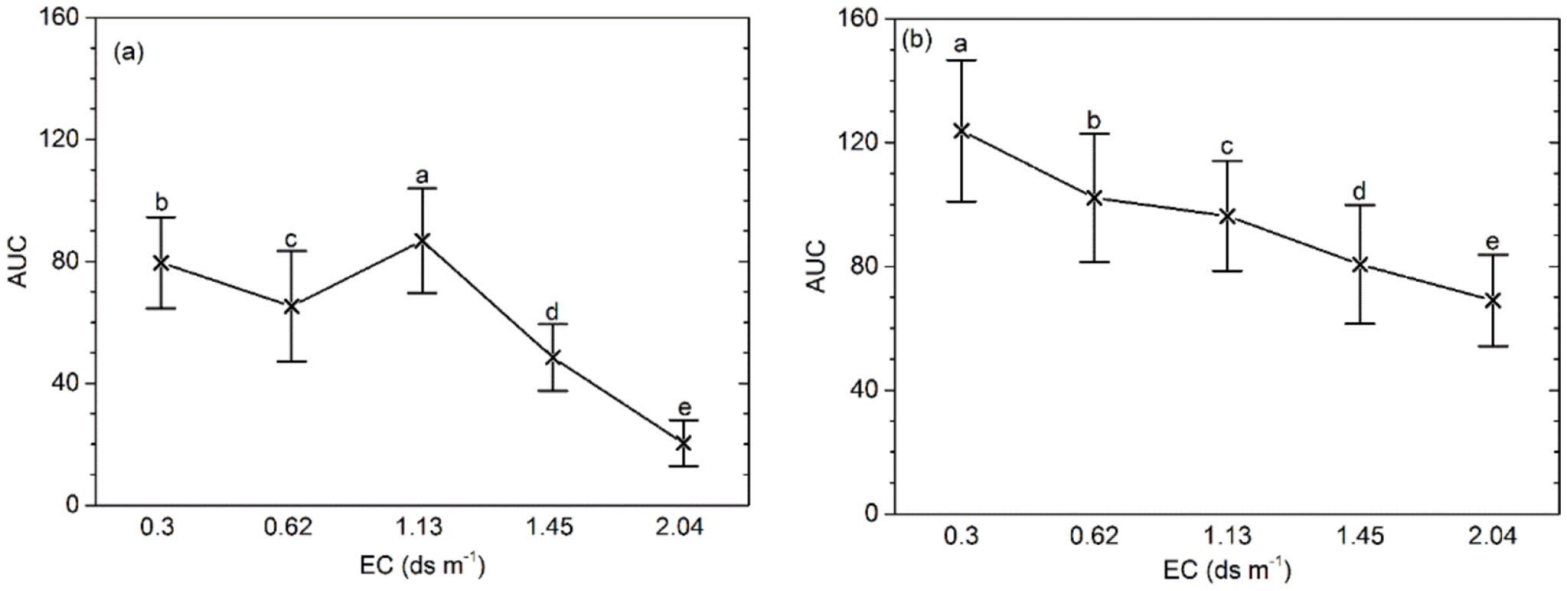
AUC of bacterial functional activity for different salinity levels, fertilizer types, and effects of the interaction between salinity and fertilizer status. **(A)** AUC of samples without organic amendment and **(B)** AUC of samples with organic amendment. Central points represent the means. Error bars represent 95% Tukey’s honestly significant differences. Different letters represent significant differences in AUC between salinity and fertilizer status.

The mean bacterial activity (AUC) at higher EC (≥ 1.45 ds m^−1^) was lower than that in the soil of EC ≤ 1.13 ds m^−1^ (*p* < 0.001). Application of organic amendment yield a lower AUC with the increasing soil salinity. In addition, at the same salinity level, the AUC of the soil samples with organic amendment was higher than that in the control (*p* < 0.0001; Figure 1). A significant interaction between salinity and the application of organic amendment was also detected.

**Figure 2:**
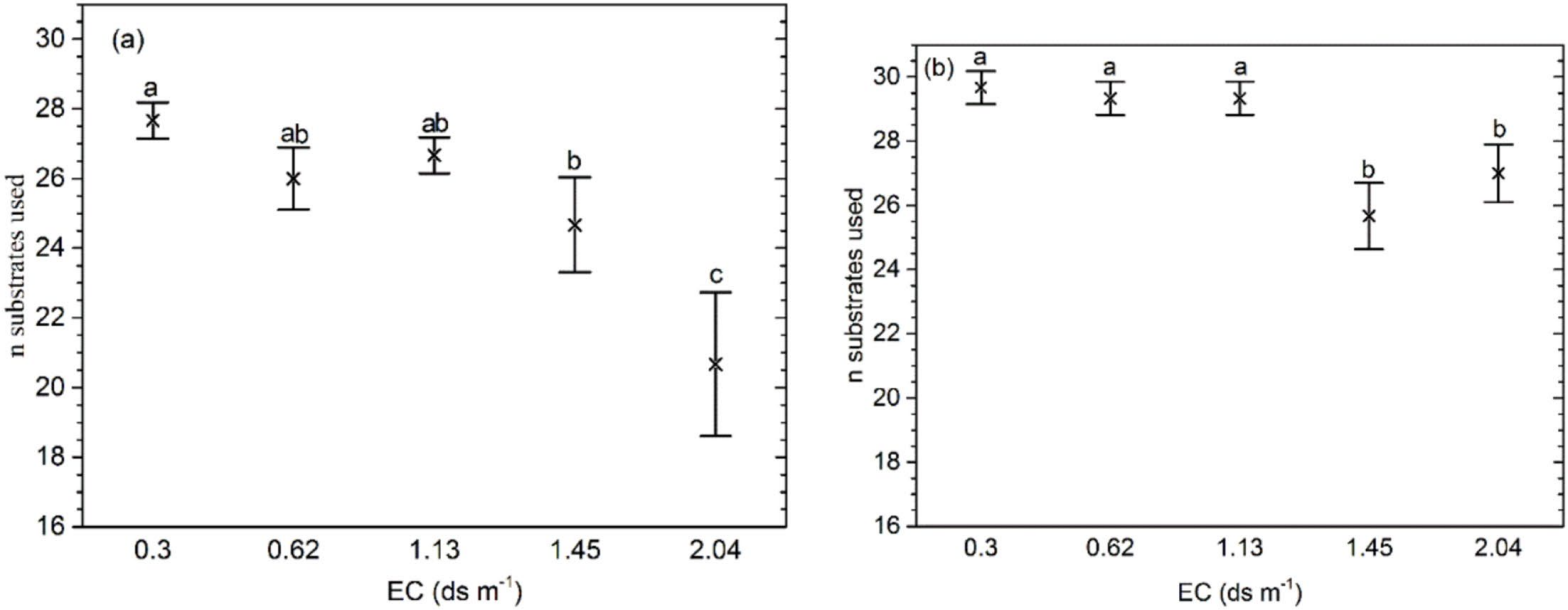
Bacteria substrates used for **(A)** soil samples without organic amendment and **(B)** those with organic amendment. The middle points represent the means. Error bars represent 95% Tukey’s honestly significant differences. Different letters represent significant differences in AUC between salinity and fertilizer status.

The mean number of substrates used on Biolog ECO MicroPlate in the soil with the highest salinity (EC 2.10 ds m^−1^) was significantly higher than in other soils (*p* = 0.000; Figure 2). For the samples with organic amendment, the mean number of substrates was significantly lower at EC ≥ 1.45 ds m^−1^ than at EC ≤ 1.13 ds m^−1^ (*p* = 0.0012). In addition, at the same salinity levels, the mean number of substrates was higher than in the soil without organic amendment. The interaction of salinity and fertilizer significantly affected the number of substrates.

The ANOSIM showed that significant differences in CLPP between salinity levels were observed in soil samples with and without organic amendment (Table 4). SIMPER analysis revealed that the largest contribution of the absence of organic amendment to the average dissimilarity was polymers among salinity groups; the bacteria of the soil of EC 2.04 ds m^−1^ soil were characterized by the highest use of polymers on Biolog ECO MicroPlate (Supplementary Table S3). Application of organic amendment corresponded to polymers and carboxylic acids, and the main consumed substrates were polymers, carboxylic acids, and carbohydrates at 0.4 ds m^−1^ (Table 5).

**Table 4:** ANOSIM on CLPP between the salinity levels studied.

Salinity	0.3 ds m^−1^	0.62 ds m^−1^	1.13 ds m^−1^	1.45 ds m^−1^	2.04 ds m^−1^
No applying fertilizer					
Global values *R* = 0.315 (0.0012)					
0.3 ds m^−1^	—	**0.846 (0.000)**	0.694 (0.0101)	0.607 (0.0212)	0.590 (0.7926)
0.62 ds m^−1^		—	**0.809 (0.000)**	0.691 (0.0128)	0.620 (0.430)
1.13 ds m^−1^			—	**0.853 (0.000)**	0.684 (0.219)
1.45 ds m^−1^				—	0.495 (0.891)
2.04 ds m^−1^					—
Applying fertilizer					
Global values *R* = 0.105 (0.0967)					
0.3 ds m^−1^	—	**0.894 (0.000)**	**0.902 (0.001)**	**0.913 (0.000)**	0.840 (0.891)
0.62 ds m^−1^		—	**0.822 (0.000)**	**0.818 (0.000)**	**0.848 (0.002)**
1.13 ds m^−1^			—	**0.855 (0.0.0108)**	0.782 (0.613)
1.45 ds m^−1^				—	0.820 (0.724)
2.04 ds m^−1^					—

The significance (*p* values) of *R* values are given. The dissimilar values are also shown in bold.

**Table 5:** Results of SIMPER analysis of dissimilarity in CLPP at different levels of salinity for soil samples with organic amendment.

Substrate chemical guild	Dissimilarity measure			Mean use				
	Average dissimilarity	Contribution %	Cumulative %	0.3 ds m^−1^	0.62 ds m^−1^	1.13 ds m^−1^	1.45 ds m^−1^	2.04 ds m^−1^
Carboxylic acids	12.105	24.21	24.21	0.23	0.23	0.24	0.26	0.25
Polymers	12.583	25.17	49.38	0.25	0.20	0.23	0.32	0.26
Carbohydrates	11.295	22.59	71.97	0.22	0.27	0.20	0.18	0.25
Miscellaneous	1.106	2.21	74.18	0.04	0.02	0.01	0.02	0.02
Amino acids	7.582	15.16	89.34	0.15	0.16	0.21	0.13	0.11
Amines	5.329	10.66	100.00	0.11	0.11	0.11	0.09	0.12

Contribution % means the proportion of a substrate group in the total effect on the dissimilarity in CLPP.

### 3.4 Shifts in microbial communities

**Figure 3:**
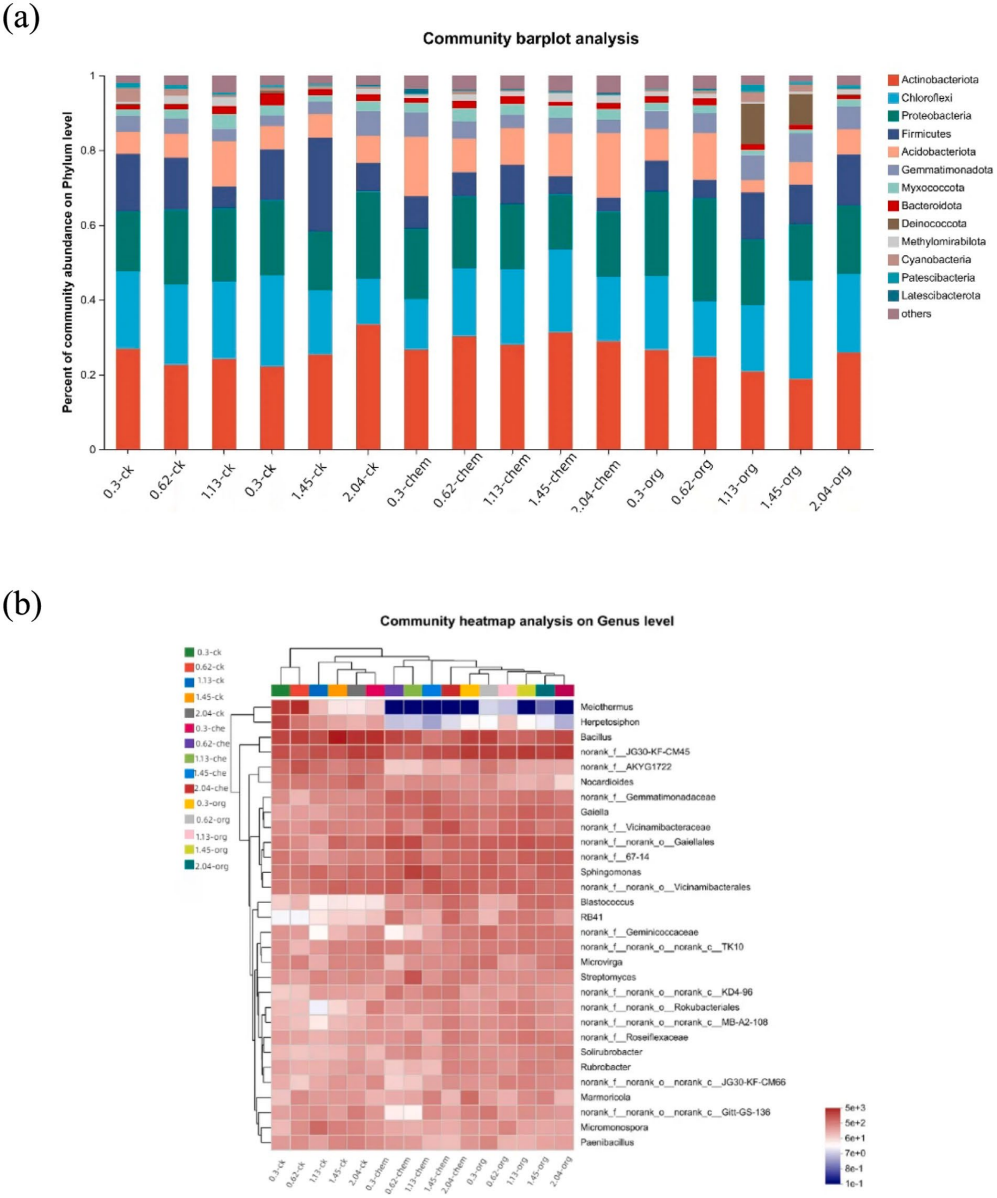
Dynamics of the bacterial community under different fertilization treatments. **(A)** Relative abundances of the top 13 phyla. **(B)** Heatmap showing the relative abundances of the top 50 genera.

The bacterial diversity of low-salinity soils was higher than high-salinity soils, and principal component analysis revealed significant changes in bacterial community structure (Supplementary Figure S1). The microbial communities structure was generally divided according to medium and low salt concentration (0–1.45 dS m^−1^) and high salt concentration (1.45–2.04 dS m^−1^). The effect of fertilization treatment on the formation of microbial community was more obvious in middle-low salt soil than in high-concentration soil. At the gate level, soil with the same salt concentration had different microbial community composition under different fertilization treatments. Actinobacteriota, Chioroflexi, Proteobacteria, and Firmicutes are the four most dominant phyla (Figure 3A). In soil with the same salinity level, the application of microbial organic fertilizer led to an increase in the proportion of Actinobacteriota and a decrease in the proportion of Chloroflexi.

In 0.3 dS m^−1^ soil, the abundance of actinomycetes increased from 23 to 27% after application of microbial organic fertilizer, while the abundance of basidiomycetes decreased from 20 to 6%. After the application of microbial organic fertilizer, RB41, blastococcus and solirubrobacter significantly increased, while Melothermus and Herpetosiphon significantly decreased. Under the same salt concentration, the actinomyces content in the treatment treated with microbial organic fertilizer was higher than that in the treatment treated with chemical fertilizer and the control group (Figure 3B). Removal of Nocardia is primarily driven by the application of microbial organic fertilizer, Nocardia being a potential human pathogen (Bao et al., 2020). The significant reduction of these genera after fertilization confirms that fertilization can reduce the environmental risks associated with salt concentration on the soil.

### 3.5 The relations among plants biomass, soil bacteria, and soil properties

For the samples without organic amendment, the CCA axes calculated for the fertilizer and soil salinity explained 66.4% (*p* = 0.0094) and 29.1% (*p* = 0.0020) of the variance (trace *p* = 0.0020). This result indicated that the soil properties (such as OM, micro-aggregate, and macro-aggregate contents) affect physiological abilities of soil microbial communities. The results of ANOSIM also revealed that there was a similarity between the CLPP and soil characteristics.

**Figure 4:**
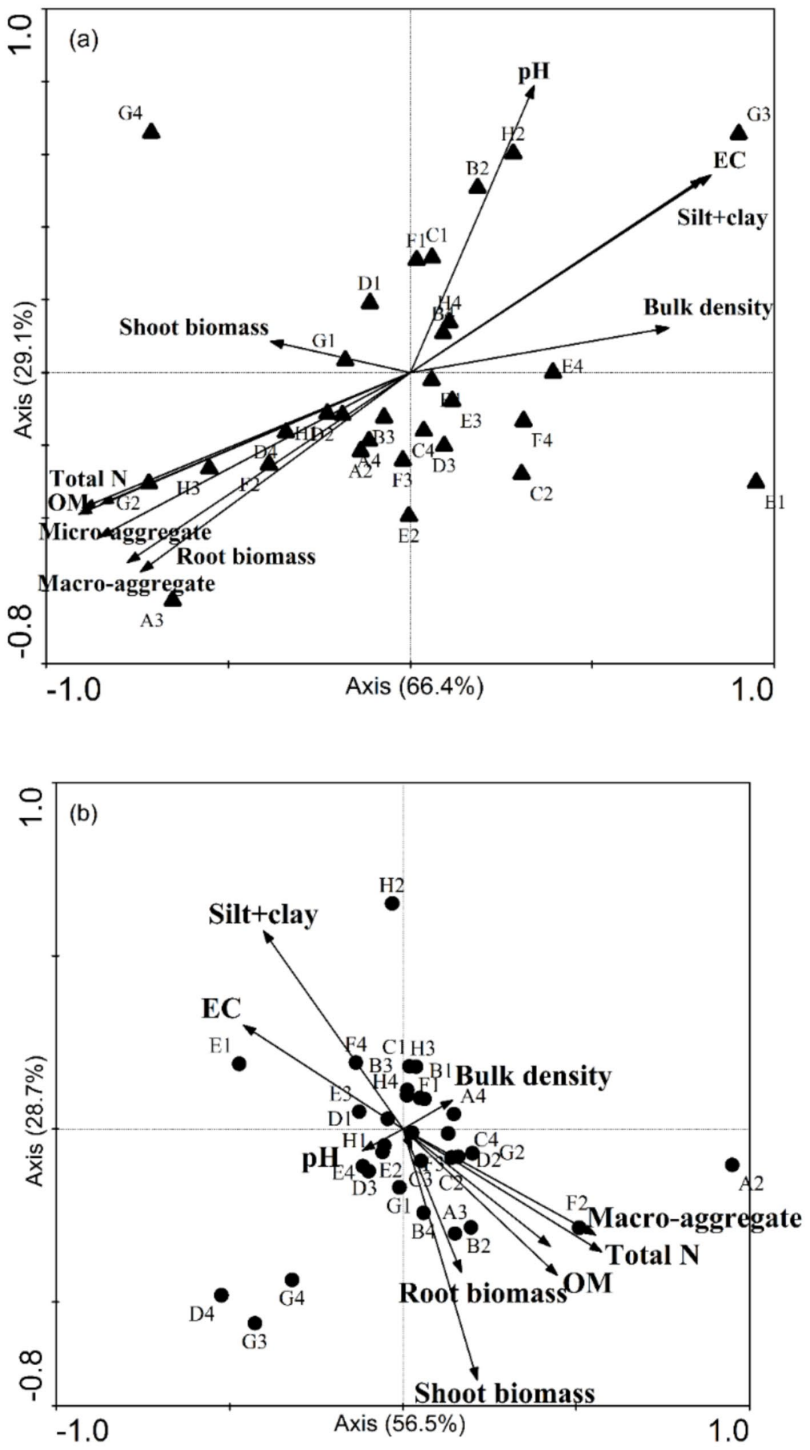
A canonical correspondence analysis (CCA) ordination plot of the bacterial substrate utilization patterns (CLPP) with soil and plant properties, including pH, total N content, organic matter (OM), bulk density, plant biomass, and the proportions of aggregates of all sizes. **(A)** The pattern without microbial fertilizer and **(B)** with microbial fertilizer. The substrate classes are indicated according to their site on a Biolog EcoPlate. A2: *β*-methyl-D-glucoside, A3: D-galactonic acid *γ*-lactone, A4: L-arginine, B1: pyruvic acid methyl ester, B2: D-xylose, B3: D-galacturonic acid, B4: L-asparagine, C1: Tween 40, C2: i-erythritol, C3: 2-hydroxy benzoic acid, C4: L-phenylalanine, D1: Tween 80, D2: D-mannitol, D3: 4-hydroxy, D4: L-serine, E1: *α*-cyclodextrin, E2: N-acetyl-D-glucosamine, E3: γ-hydroxybutyric acid, E4: L-threonine, F1: glycogen, F2: D-glucosaminic acid, F3: itaconic acid, F4: glycyl-L-glutamic acid, G1: D-cellobiose, G2: glucose-1-phosphate, G3: α-ketobutyric acid, G4: phenylethyl-amine, H1: α-D-lactose, H2: D,L-α-glycerol phosphate, H3: D-malic acid, H4: putrescine.

CCA indicated a strong negative relation between the use of D-galactonic acid *γ*-lactone and root biomass (Figure 4A). The use of D-malic acid, D-glucosaminic acid, and glucose-1-phosphate was negatively related to soil total N, microaggregate content (53–250 μm), macro-aggregate content (> 250 μm), and OM content. In addition, it was obvious that the use of D-, L-*α*-glycerol phosphate, and D-xylose had a strong relation with soil pH.

For soil samples with organic amendment, the CCA axis was also significant (*p* = 0.041). Nevertheless, it was different for the relation between soil properties and microbial characteristics.

The CCA analysis showed a positive correlation between the silt + clay fraction and D-, L-α-glycerol phosphate and glycyl-L-glutamic acid. Shoot biomass, total N, OM content, and the proportion of macro-aggregates (> 250 μm) had a strong relation with *β*-methyl-D-glucoside, D-xylose, and D-glucosaminic acid (Figure 4B).

## 4 Discussion

In this study, we found that the response of microbial activity to application of organic amendment depends on the soil salinity. It had a significant effect on soil characteristics and on diversity of the soil microbial community in soil samples of lower salinity with application of organic amendment. Furthermore, there was a significant relation between the soil characteristics and plant biomass and composition and functional soil bacterial diversity, which was measured with Biolog ECO MicroPlate.

### 4.1 The response of physicochemical properties, plant biomass and soil bacteria to application of organic amendment in soils of different salinity

In the soil of EC ≥ 1.45 ds m^−1^, the root and shoot biomass were obviously lower than in the soil of EC ≤ 1.13 ds m^−1^. At the same salt concentration, application of organic amendment increased the plant biomass compared with soil samples without organic amendment (Table 3). Plant residues in soil and root exudation are important input pathways of the available substrates (Chowdhury et al., 2011). Thus, the application of organic amendment may improve the ability of the microbial community to utilize soil resources. Soils of different salinity and fertilizer status showed diverse properties. With the increasing salinity, the proportion of macro-aggregates, total N, and OM content decreased, whereas the silt + clay fraction increased (Table 3). The application of organic amendment narrowed the ranges of those factors influenced by salinity. Similar findings were observed on the North China Plain after long-term application of organic manure (Yu et al., 2012): Organic amendment increased the mass proportion of macro-aggregates at the expense of micro-aggregates and the silt + clay fraction. Our results also suggest that soil salt concentration and organic amendment can affect the turnover rate of soil nutrients and C cycling. Soil microorganisms can produce decomposing enzymes to improve the release of nutrients (Hu et al., 2017). The addition of microbes to the fertilizer may strongly enhance soil OM mineralization and the formation of a new microbial structure.

In our study, soil bacterial activity can be demonstrated by AUC measured by means of Biolog ECO MicroPlate. It differed between salinity levels and fertilizer groups. The mean bacterial activity (AUC) at higher EC (≥1.45 ds m^−1^) was lower than that in the soil of EC ≤ 1.13 ds m^−1^. At the same salinity level, the AUC of the soil samples with organic amendment was higher than that of the control (Figure 1). Sodicity can increase swelling, dispersion, and slaking upon wetting and then alters physical properties of the soil and microbial community structure and functions (Wong et al., 2009). In our study, CLPP differed between soil salinity levels with organic amendment and without (Table 5). Schultz and Ducklow (2000) reported that there is a decrease in the bacterial activity from fresh water to saltwater. They researched the stability of bacterial composition and activity at different salinity levels (They et al., 2013). He found that CLPP are different between limnetic-oligohaline and mesohaline. Troussellier and Giorgio using a culture-independent method, demonstrated that the activity of bacterial communities decreases along the salinity gradient (Troussellier et al., 2002; Giorgio and Bouvier, 2002).

Biolog substrate utilization patterns revealed that the microbial metabolic potential and functional diversity declined with the increasing soil salt concentration (Figures 1, 2). Sardinha found that the number of cells of prokaryotic microorganisms decreases significantly with increasing salinity (Sardinha et al., 2003). The microbial community structure also shifted as a consequence of changes in salinity. Pankhurst reported that fungi are more affected by salinization (Pankhurst et al., 2001), and Badran reached the same conclusion by phospholipid fatty acid (PLFA) analysis (Ahmed et al., 2018). Sardinha found that the ratio of fungi in microbial biomass is 90 and 17% repectively when the salt content is 2.2 and 13.2 mg g^−1^ soil (Sardinha et al., 2003). In a saline soil, the composition of the bacterial community can undergo a shift (Chika et al., 2022; Ma et al., 2016). In addition, the fungal cell membranes may be influenced (Neelavar et al., 2017). The possible reason for our results is that salinization had an adverse effect on vegetation growth, and the amount of plant residues in the soil decreased. The need of saprotrophic microfungi for energy was not fully met.

Our data suggest that the soil MBC decreased with the increase in salt concentration. When the salt concentration was lower than 1.13 ds m^−1^ at the early stage of application of organic amendment, the MBC increased significantly as compared to the control (Supplementary Figure S2). Compared to the soil samples without organic amendment, metabolizable C and microbial biomass increased after application of organic amendment. Mavi reported that the addition of C can improve the microbial tolerance of salinity (Rui et al., 2021). Wichern also demonstrated that salinity strongly reduces microbial biomass and inhibits substrate decomposition (Wichern et al., 2006). Analysis of the combined effects of salinity and low pH leads to the conclusion that salinization has a suppressive effect on microbial biomass (Wong et al., 2009). Garcia also found that soil respiration is inhibited in a degraded high-EC soil (Otieno et al., 2018). Four months after application of organic amendment, the MBC was less than that at the early stage. Most likely, there was a shift in community structure, from the one dominated by fungi to the structure dominated by halotolerant bacteria consisting mainly of bacteria that are less active, competitive, and diverse (Pankhurst et al., 2001).

The increase in the soil salt concentration decreased the diversity of the soil microbial community. The richness and Shannon’s index decreased, whereas the Simpson index increased as the soil salt concentration increased (Figure 2; Table 2). The addition of organic amendment increased the diversity of the soil microbial community as compared to the soil samples without organic amendment at the same salt concentration. Application of organic amendment also changed the type and magnitude of substrate utilization. A Biolog ECO MicroPlate contains six kinds of substrates (chemical guilds): amino acids, carbohydrates, carboxylic acids, miscellaneous, amines, and polymers (Klimek et al., 2016). The mean utilization rates of mainly C sources changed with the increasing salinity and after application of organic amendment (Table 5). Min also found that irrigation with water (change in salinity) can alter soil microbial activity and decrease community functional diversity (Min et al., 2015). In our saline soils, organic amendment increased microbial activity and diminished the adverse effects of salinity on soil microbial biomass and community diversity in soil samples (Figure 1; Supplementary Figure S2). Zhong found that application of organic manure significantly increases the functional diversity as compared to treatments without organic manure (Zhong et al., 2010). Besides, in the maintenance of biodiversity, root exudates play an important role. Adekunle emphasized the importance of organic C in microbial population and diversity (Adekunle et al., 2005). In addition, application of organic amendment increased the soil microbial biomass and community diversity by providing more of organic C. Zhong reported that the effects of organic amendment and organic manure on a soil microbial community and diversity are different according to an analysis by the bacteria sequencing technology (Zhong et al., 2010). Horn also reported that the microbial communities can undergo a rapid change in response to the additon of organic resources along a salinity gradient (Horn et al., 2014).

Zahran found that there are differences in the cell morphology among soils of different salinity (Sharma et al., 2021). Generally, the cells enlarge with the increasing salinity. The interaction of these factors may be one of the reasons for the shift in soil microbial biomass and community diversity. Some showed that the priming effect is caused by organic amendment and is related to the reproductive strategy (Cornwell et al., 2006). Paterson and Sim (2013) reported that priming effect is not a ubiquitous function of all components of microbial communities, and addition of labile C may increase the growth and activity of r-strategist microbial communities.

### 4.2 The relation between an array of microbiological and soil characteristics and plant biomass after organic amendment applied in saline soils

The change in soil microorganism community structure and function between salinity levels can determine soil characteristics. The ANOSIM showed that there were significant differences in CLPP between salinity levels (Table 4). They also obtained similar results on bacterial composition and activity in different salinity ranges (They et al., 2013). In addition, Zhao reported that salinity is a major driving force behind bacterial diversity according to data on 16S rRNA sequences (Zhao et al., 2018). Our experiment revealed that higher salinity soil showed lower respiration rates (Supplementary Figure S3). This result is consistent with other studies (Wichern et al., 2006). The reason is that high salt concentration cause a low osmotic potential. Small microbial cells may result from drawing of water out of the cells (Yan and Marschner, 2012). Nevertheless, the results of Rousk and Asghar contradict ours (Klimek et al., 2016; Asghar et al., 2012). They found that the response of soil respiration was not different significantly at different EC levels in the short-term. One possible reason is the difference in soil types and environment conditions between the studies. Application of organic amendment significantly increased soil CO_2_ emissions (Supplementary Table S4). The effect decreased with the increasing soil salinity (Supplementary Figure S2). López-Valdez and Franco-Hernández demonstrated that when microorganisms are added along with sludge, they accelerate OM decomposition in a saline soil (López-Valdez et al., 2010; Franco-Hernández et al., 2003).

Our results of CCA indicate that soil pH, OM content, root biomass, and proportions of micro-aggregates and macro-aggregates had a strong relation with bacterial CLPP. In contrast, for soil samples with organic amendment, the silt + clay fraction instead of soil pH and shoot biomass instead of root biomass became the indictors closely tracking CLPP. The shoot-to-root biomass partitioning largely depends upon plant C: N content. Shoot biomass is significantly greater in soils subjected to high-N treatment (Landry Rossdeutsch et al., 2020). Soil nutrients (N, P, and S) are released into the soil through mineralization. Thus, soil microorganisms have a strong relation with root biomass. Yu et al. (2022) reported that intra-aggregate OM is incorporated into (and physically stabilized in) macro-aggregates. We found that application of organic amendment could increases the proportion of macro-aggregate and decreases the silt + clay fraction. We also found that the soil organic C in the silt + clay fraction after application of organic amendment was larger than in the controls. In addition, soil pH serves an important function in the physical condition of microorganism (Figure 3A). Nevertheless, the application of organic amendment diminished the effect of pH (Figure 3B). This finding suggested that the application of organic amendment changed the microbial community structure and function. The influence of the silt + clay fraction on soil microbial community increased. OM content and total N affect the microbial activity according to the supply of energy by assimilation and stimulate the reproduction of soil microorganism (Bardgett and Wardle, 2010). The stability of micro-aggregate and macro-aggregate is an important factor for analysis of soil structure. Microbial acitivity has an effect on soil crumb structure, and soil structural properties also affect the microbial distribution and biomass. For soil samples with organic amendment, there are six chemical substrates in Biolog ECO MicroPlate. Polymers contributed the most to the differences in CLPP between salinity levels (Table 5). Compared to the samples without organic amendment, the change in the contribution of substrates and the relation between environment factors and substrates suggested that the addition of fertilizer altered the microbial community. Wu found that the application of organic amendment to a saline soil can elevate the abundance of soil organisms and soil nutrient content (Hou et al., 2021). In our soil samples with organic amendment, soil OM content, total N, the proportion of macro-aggregates and the silt + clay fraction positively correlated with the number of soil organisms. The tight relation between soil properties and organisms can regulate the structure and functioning of an ecosystem at the community level (Wardle et al., 2004).

The limitations of microbial organic fertilizers mainly include issues related to viability and the sustainability of their effects. On one hand, microbial organic fertilizers are prone to viability issues during production, transportation, and storage. On the other hand, microbial organic fertilizers do not match the long-lasting effects of chemical fertilizers, requiring prolonged application, thus increasing economic costs.

## 5 Conclusion

We found that the microbial metabolic potential and functional diversity decline with the increasing soil salinity. For the soil of EC ≤ 1.13 ds m^−1^, application of organic amendment improves activity of the microbial community and microbial biomass. We also demonstrated that soil salt concentration and organic amendment affect soil physicochemical properties and the relation between the environment and microbial community. Our results are potentially relevant for amelioration of the saline-alkali land in the Yellow River Delta, China. This study provides a strong theoretical basis for using microbial organic fertilizers to improve saline-alkali soil.

## Data Availability

The original contributions presented in the study are included in the article/Supplementary material, further inquiries can be directed to the corresponding author.

## Footnotes

^1^ http://www.biolog.com

^2^ http://drive5.com/usearch/manual/singletons.html

## References

[ref1] Adekunle V. A. J. Dafiewhare H. B. Ajibode OF (2005). Microbia population and diversity as influenced by soil pH and organic matter in different forest ecosystems. Pakistan J. Biol. Sci. 8, 1478–1484. doi: 10.3923/pjbs.2005.1478.1484

[ref2] Adesemoye A. O. Torbert H. A. Kloepper J. W. (2008). Enhanced plant nutrient use efficiency with PGPR and AMF in an integrated nutrient management system. Can. J. Microbiol. 54, 876–886. doi: 10.1139/W08-081, PMID: 18923557

[ref3] Adnane B. Karim L. Mohamed C. Youssef Z. Driss D. (2018). Soil microbial resources for improving fertilizers efficiency in an integrated plant nutrient management system. Front. virol. 9, 1–25. doi: 10.3389/fmicb.2018.01606, PMID: 30108553 PMC6079243

[ref4] Ahmed M. el-Zayat S. el-Sayed M. (2018). Cellulolytic activity of cellulose-decomposing fungi isolated from Aswan hot desert soil, Egypt. J. Biol. Stud. 1, 35–48. doi: 10.62400/jbs.v1i2.9

[ref5] Asghar H. N. Setia R. Marschner P. (2012). Community composition and activity of microbes from saline soils and non-saline soils respond similarly to changes in salinity. Soil Biol. Biochem. 47, 175–178. doi: 10.1016/j.soilbio.2012.01.002

[ref9001] Blake G. R. (1965). Methods of soil analysis: Part 1 physical and mineralogical properties, including statistics of measurement and sampling. The American Society of Agronomy, Inc. 37, 2055–2064. doi: 10.2134/agronmonogr9.1.c30

[ref6] Bao J. Wang X. Gu J. Dai X. Zhang K. Wang Q.. (2020). Effects of macroporous adsorption resin on antibiotic resistance genes and the bacterial community during composting. Bioresour. Technol. 295:121997. doi: 10.1016/j.biortech.2019.121997, PMID: 31634802

[ref7] Bardgett R. D. Wardle D. A. (2010). Aboveground–Belowground Linkages. New York: Oxford University Press.

[ref8] Benizri E. Amiaud B. (2005). Relationship between plants and soil microbial communities in fertilized grasslands. Soil Biol. Biochem. 37, 2055–2064. doi: 10.1016/j.soilbio.2005.03.008

[ref9] Chika J. U. Fabrizio C. Adhikari J. S. Shen Y. X. Badireddy A. R. Mouser P. J. (2022). Salinity and hydraulic retention time induce membrane phospholipid acyl chain remodeling in *Halanaerobium congolense* WG10 and mixed cultures from hydraulically fractured shale wells. Front. Microbiol. 13, 1–14. doi: 10.3389/fmicb.2022.1023575 PMC9687094 36439785

[ref10] Chowdhury N. Marschner P. Burns R. (2011). Response of microbial activity and community structure to decreasing soil osmotic and matric potential. Plant Soil 344, 241–254. doi: 10.1007/s11104-011-0743-9

[ref11] Cornwell R. E. Law Smith M. J. Boothroyd L. G. Moore F. R. Davis H. P. Stirrat M.. (2006). Reproductive strategy, sexual development and attraction to facial characteristics. Philos. Trans. R. Soc. Lond. Ser. B Biol. Sci. 361, 2143–2154. doi: 10.1098/rstb.2006.1936, PMID: 17118929 PMC1764838

[ref12] Daei G. Ardekani M. R. Rejali F. Teimuri S. Miransari M. (2009). Alleviation of salinity stress on wheat yield, yield components, and nutrient uptake using arbuscular mycorrhizal fungi under field conditions. J. Plant Physiol. 166, 617–625. doi: 10.1016/j.jplph.2008.09.013, PMID: 19100656

[ref13] Elgharably A. Marschner P. (2011). Microbial activity and biomass and N and P availability in a saline sandy loam amended with inorganic N and lupin residues. Eur. J. Soil Biol. 47, 310–315. doi: 10.1016/j.ejsobi.2011.07.005

[ref14] Encarnación F. Arunava P. Cláudia P. Juan R. Daniela B. Francisco J. M.. (2021). Elevated temperature may reduce functional but not taxonomic diversity of fungal assemblages on decomposing leaf litter in streams. Glob. Chang. Biol. 28, 115–127. doi: 10.1111/gcb.15931, PMID: 34651383

[ref15] Franco-Hernández O. Mckelligan-González A. N. López-Olguin A. M. Espinosa-Ceron F. Escamilla-Silva E. Dendooven L. (2003). Dynamics of carbon, nitrogen and phosphorus in soil amended with irradiated, pasteurized and limed biosolids. Bioresour. Technol. 87, 93–102. doi: 10.1016/S0960-8524(02)00188-8, PMID: 12733582

[ref16] Gabriela W. Monika M. Jacek K. Łukasz R. Joanna C. Jaco V.. (2023). How important are the relations between vegetation diversity and bacterial functional diversity for the functioning of novel ecosystems? Sustain. For. 15, 2–16. doi: 10.3390/su15010678, PMID: 39659294

[ref17] Giorgio P. A. D. Bouvier T. C. (2002). Linking the physiologic and phylogenetic successions in free-living bacterial communities along an estuarine salinity gradient. Limnol. Oceanogr. 47, 471–486. doi: 10.4319/lo.2002.47.2.0471

[ref18] Gömöryová E. Ujházy K. Martinák M. Gömöry D. (2013). Soil microbial community response to variation in vegetation and abiotic environment in a temperate old-growth forest. Appl. Soil Ecol. 68, 10–19. doi: 10.1016/j.apsoil.2013.03.005, PMID: 39678304

[ref19] Hammer E. C. Nasr H. Wallander H. (2011). Effects of different organic materials and mineral nutrients on arbuscular mycorrhizal fungal growth in a Mediterranean saline dryland. Soil Biol. Biochem. 43, 2332–2337. doi: 10.1016/j.soilbio.2011.07.004

[ref20] Hannah L. B. Nicola J. D. Bradley S. C. Gavin L. (2021). Measuring change in biological communities: multivariate analysis approaches for temporal datasets with low sample size. Peer J. 9, 2–38. doi: 10.7717/peerj.11096/PMC8038644 33889442

[ref21] Hasbullah H. Marschner P. (2015). Residue properties influence the impact of salinity on soil respiration. Biol. Fert. Soils 51, 99–111. doi: 10.1007/s00374-014-0955-2

[ref22] Horn D. J. V. Okie J. G. Buelow H. N. Gooseff M. N. Barrett J. E. (2014). Takacs-Vesbach CD (2014) soil microbial responses to increased moisture and organic resources along a salinity gradient in a Polar Desert. Appl. Environ. Microb. 80, 3034–3043. doi: 10.1128/AEM.03414-13, PMID: 24610850 PMC4018898

[ref23] Hou Y. Zeng W. Hou M. Wang Z. Luo Y. Lei G.. (2021). Responses of the soil microbial community to salinity stress in maize fields. Biology 10, 1–13. doi: 10.3390/biology10111114, PMID: 34827107 PMC8614889

[ref24] Hu H. Chao J. Wu Y. P. Cheng Y. X. (2017). Bacterial and fungal communities and contribution of physicochemical factors during cattle farm waste composting. Microbiol 6, 1–11. doi: 10.1002/mbo3.518 PMC5727367 28736905

[ref25] Jin Z. Lei J. Li S. Xu X. (2015). Metabolic characteristics of microbial communities of Aeolian sandy soils induced by saline water drip irrigation in shelter forests. Eur. J. Soil Sci. 66, 476–484. doi: 10.1111/ejss.12230

[ref26] Ju F. Zhang T. (2015). Bacterial assembly and temporal dynamics in activated sludge of a full-scale municipal wastewater treatment plant. ISME J. 9, 683–695. doi: 10.1038/ismej.2014.162, PMID: 25180966 PMC4331583

[ref27] Klimek B. Chodak M. Jaźwa M. Solak A. Tarasek A. Niklińska M. (2016). The relationship between soil bacteria substrate utilisation patterns and the vegetation structure in temperate forests. Eur. J. Forest Res. 135, 179–189. doi: 10.1007/s10342-015-0929-4

[ref28] Klimek B. Niklińska M. Jaźwa M. Tarasek A. Tekielak I. Musielok Ł. (2015). Covariation of soil bacteria functional diversity and vegetation diversity along an altitudinal climatic gradient in the Western Carpathians. Pedobiologia 58, 105–112. doi: 10.1016/j.pedobi.2015.04.005

[ref29] Klopp H. W. Daigh A. L. M. (2020). Measured saline and sodic solutions effects on soil saturated hydraulic conductivity, electrical conductivity and sodium adsorption ratio. Arid Land Res. Manag. 34, 264–286. doi: 10.1080/15324982.2019.1672221, PMID: 39678644

[ref30] Koner S. Chen J. S. Hsu B. M. Tan C. W. Fan C. W. Chen T. H.. (2021). Assessment of carbon substrate catabolism pattern and functional metabolic pathway for microbiota of limestone caves. Microorganisms 9, 2–16. doi: 10.3390/microorganisms9081789 PMC8398112 34442868

[ref31] Landry Rossdeutsch R. Schreiner P. Skinkis P. A. Deluc L. (2020). Nitrate uptake and transport properties of two grapevine rootstocks with varying vigor. Front. Plan. Sci. 11:2020. doi: 10.3389/fpls.2020.608813 PMC7847936 33537044

[ref32] López-Valdez F. Fernández-Luqueño F. Luna-Guido M. L. Marsch R. Olalde-Portugal V. Dendooven L. (2010). Microorganisms in sewage sludge added to an extreme alkaline saline soil affect carbon and nitrogen dynamics. Appl. Soil Ecol. 45, 225–231. doi: 10.1016/j.apsoil.2010.04.009

[ref33] Ma X. Liu M. Li Z. (2016). Shifts in microbial biomass and community composition in subtropical paddy soils under a gradient of manure amendment. Biol and Fert Soils 52, 775–787. doi: 10.1007/s00374-016-1118-4

[ref34] Min W. Guo H. Zhang W. Zhou G. Ma L. Ye J.. (2015). Response of soil microbial community and diversity to increasing water salinity and nitrogen fertilizer rate in an arid soil. Soil Plant. Sci. 66, 117–126. doi: 10.1080/09064710.2015.1078838

[ref35] Neelavar S. R. Liliya G. N. Felix B. (2017). Pellet size affects mycelial ergosterol content in aquatic hyphomycetes. Mycologia 96, 182–201. doi: 10.1080/15572536.2005.11832983 21148860

[ref36] Otieno J. R. Kamau E. M. Oketch J. M. Ngoi J. M. Gichuki A. M. Binter S.. (2018). Whole genome analysis of local Kenyan and global sequences unravels the epidemiological and molecular evolutionary dynamics of RSV genotype ON1 strains. Virus Evolution 68, 5448–5459. doi: 10.1093/ve/vey027 PMC6153471 30271623

[ref37] Pankhurst C. E. Yu S. Hawke B. G. (2001). Harch BD (2001) capacity of fatty acid profiles and substrate utilization patters to describe differences in soil microbial communities associated with increased salinity or alkalinity at three locations in South Australia. Biol. Fert. Soils 33, 204–217. doi: 10.1007/s003740000309

[ref38] Paterson E. Sim A. (2013). Soil-specific response functions of organic matter mineralization to the availability of labile carbon. Global Chang. Biol. 19, 1562–1571. doi: 10.1111/gcb.12140, PMID: 23505211

[ref39] Rackley S. (2023). Afforestation and other land- and soil-based methods. Elservier: Negative Emissions Technologies for Climate Change Mitigation.

[ref40] Rui H. S. Yong X. Y. Chao R. G. Huai Y. Y. (2021). Soil texture alters the impact of salinity on carbon mineralization. Agronomy 11, 2–13. doi: 10.3390/agronomy11010128, PMID: 39659294

[ref41] Sardinha M. Müller T. Schmeisky H. Joergensen R. G. (2003). Microbial performance in soils along a salinity gradient under acidic conditions. Appl. Soil Ecol. 23, 237–244. doi: 10.1016/S0929-1393(03)00027-1

[ref42] Schultz G. E. Ducklow H. (2000). Changes in bacterioplankton metabolic capabilities along a salinity gradient in the New York river estuary, Virginia, USA. Aquat. Microb. Ecol. 22, 163–174. doi: 10.3354/ame022163

[ref43] Setia R. Marschner P. Baldock J. Chittleborough D. (2010). Is CO_2_ evolution in saline soils affected by an osmotic effect and calcium carbonate? Biol. Fertil. Soils 46, 781–792. doi: 10.1007/s00374-010-0479-3

[ref44] Sharma A. Dev K. Sourirajan A. Choudhary M. (2021). Isolation and characterization of salt-tolerant bacteria with plant growth-promoting activities from saline agricultural fields of Haryana, India. J. Genetic Eng. Biotechnol. 19, 99–19. doi: 10.1186/s43141-021-00186-3 PMC8239113 34181159

[ref45] Somak C. Markus L. Ashish A. M. Timothy G. Jianbei H. Robert I. G.. (2022). Plants with arbuscular mycorrhizal fungi efficiently acquire nitrogen from substrate additions by shaping the decomposer community composition and their net plant carbon demand. Plant Soil 475, 473–490. doi: 10.1007/s11104-022-05380-x, PMID: 39679138

[ref46] Songlin W. Wei F. Matthias C. Baodong C. Yong G. Z. Longbin H. (2024). Soil organic matter dynamics mediated by arbuscular mycorrhizal fungi – an updated conceptual framework. New Phytol. 242, 1417–1425. doi: 10.1111/nph.19178, PMID: 37529867

[ref47] Temesgen K. Dereje D. Ararsa B. (2023). The effect of organic solid waste compost on soil properties, growth, and yield of Swiss chard crop (*Beta vulgaris* L.). Sci. World J. 2023, 1–10. doi: 10.1155/2023/6175746, PMID: 37908492 PMC10615583

[ref48] They N. H. Ferreira L. M. H. Marins L. F. Abreu P. C. (2013). Stability of bacterial composition and activity in different salinity waters in the dynamic Patos lagoon estuary: evidence from a Lagrangian-like approach. Microbial Ecol 66, 551–562. doi: 10.1007/s00248-013-0259-3, PMID: 23812105

[ref49] Troussellier M. H. Schäfer N. Batailler L. Bernard C. Courties P. Lebaron G.. (2002). Bacterial activity and genetic richness along an estuarine gradient (Rhone river plume, France). Aquat. Microb. Ecol. 28, 13–24. doi: 10.3354/ame028013

[ref50] Wardle D. A. Bardgett R. D. Klironomos J. N. Setälä H. van der Putten W. H. Wall D. H. (2004). Ecological linkages between aboveground and belowground biota. Science 304, 1629–1633. doi: 10.1126/science.1094875, PMID: 15192218

[ref51] Wichern J. Wichern F. Joergensen R. G. (2006). Impact of salinity on soil microbial communities and the decomposition of maize in acidic soils. Geoderma 137, 100–108. doi: 10.1016/j.geoderma.2006.08.001

[ref52] Wong V. N. L. Greene R. S. B. Dalal R. C. Murphy B. W. (2009). Soil carbon dynamics in saline and sodic soils: a review. Soil Use Manag. 26, 2–11. doi: 10.1111/j.1475-2743.2009.00251.x

[ref53] Xiang Y. S. Wei J. L. Yu F. H. Long L. X. Kun K. F. Yan Y. Z.. (2023). Ecosystem multi functionality and soil microbial communities in response to ecological restoration in an alpine degraded grassland. Front. plant Sci 24, 1–12. doi: 10.3389/fpls.2023.1173962 PMC10431941 37593047

[ref54] Xuekai W. Xin X. C. Han L. Linna G. Yanli L. Liu X.. (2021). Effects of Lactic Acid Bacteria on Microbial Metabolic Functions of Paper Mulberry Silage: A BIOLOG ECO Microplates Approach. Front. Microbiol. 12, 1–9. doi: 10.3389/fmicb.2021.689174 PMC8267872 34248912

[ref55] Yan N. Marschner P. (2012). Response of microbial activity and biomass to increasing salinity depends on the final salinity, not the original salinity. Soil Biol. Biochem. 53, 50–55. doi: 10.1016/j.soilbio.2012.04.028

[ref57] Yu H. Ding W. Luo J. Geng R. Ghani A. Cai Z. (2012). Effects of long-term compost and fertilizer application on stability of aggregate-associated organic carbon in an intensively cultivated sandy loam soil. Biol. Fert. Soils 48, 325–336. doi: 10.1007/s00374-011-0629-2

[ref58] Yu W. J. Huang W. J. Weintraub-Leff S. R. Hall S. J. (2022). Where and why do particulate organic matter (POM) and mineral-associated organic matter (MAOM) differ among diverse soils? Soil Biol. Biochem. 172, 108756–108718. doi: 10.1016/j.soilbio.2022.108756

[ref59] Zhao J. Sun X. Awasthi M. Wang Q. Ren X. Li R.. (2018). Performance evaluation of gaseous emissions and Zn speciation during Zn-rich antibiotic manufacturing wastes and pig manure composting. Bioresour. Technol. 267, 688–695. doi: 10.1016/j.biortech.2018.07.088, PMID: 30071460

[ref60] Zhong W. Gu T. Wang W. Zhang B. Lin X. Huang Q.. (2010). The effects of mineral fertilizer and organic manure on soil microbial community and diversity. Plant Soil 326, 511–522. doi: 10.1007/s11104-009-9988-y

