# Supplementary material for: The response of soil microbial community to application of organic amendment to saline land
Source: Front Microbiol. 2025 Jan 6;15:1481156. doi: 10.3389/fmicb.2024.1481156 (PMC11743944; doi:10.3389/fmicb.2024.1481156)
Supplement: Supplementary file 1 [file Data_Sheet_1.pdf]

The response of soil microbial community to application of organic amendment to saline land

Peifei Cong[a,b,c], Pengfei Huang[a*], Zhisheng Huang[b,d,e,f]

[a] Institute of Farmland Irrigation, Chinese Academy of Agricultural Sciences, Xinxiang 453002, China

[b]DeepBlue Academy of Sciences

[c]Yucheng Comprehensive Experiment Station, Key Laboratory of Ecosystem Network Observation and Modeling, Institute of Geographic Sciences and Natural Resources Research, Chinese Academy of Sciences, Beijing 100101, China

[d] Clinical Research Center for Mental Disorders, Shanghai Pudong New Area Mental Health Center, Tongji University School of Medicine, China

[e]Knowledge Representation and Reasoning (KR&R) Group, Vrije Universiteit Amsterdam, Amsterdam, The Netherlands

[f]Haoxinqing Health Industry Group, Beijing, China

* Correspondence: huangpengfei@caas.cn

Table S1 Carbon Source in EcoPlate^{TM}

A1<br>Water	A2<br>ß-Methyl-D-Glucoside	A3<br>D-Galactonic Acid y-Lactone	A4<br>L-Arginine	A1<br>Water	A2<br>ß-Methyl-D-Glucoside	A3<br>D-Galactonic Aci y-Lactone	A4<br>L-Arginine	A1<br>Water	A2<br>ß-Methyl-D-Glucoside	A3<br>D-Galactonic Acid y-Lactone	A4<br>L-Arginine
B1<br>Pyruvic Acid Methyl Ester	B2<br>D-Xylose	B3<br>D-Galacturonic Acid	B4<br>L-Asparagine	B1<br>Pyruvic Acid Methyl Ester	B2<br>D-Xylose	B3<br>D-Galacturonic Acid	B4<br>L-Asparagine	B1<br>Pyruvic Acid Methyl Ester	B2<br>D-Xylose	B3<br>D-Galacturonic Acid	B4<br>L-Asparagine
C1<br>Tween 40	C2<br>I-Erythritol	C3<br>2-Hydroxy Benzoic Acid	C4<br>L-Phenylalanine	C1<br>Tween 40	C2<br>I-Erythritol	C3<br>2-Hydroxy Benzoic Acid	C4<br>L-Phenylalanine	C1<br>Tween 40	C2<br>I-Erythritol	C3<br>2-Hydroxy Benzoic Acid	C4<br>L-Phenylalanine
D1<br>Tween 80	D2<br>D-Mannitol	D3<br>4-Hydroxy Benzoic Acid	D4<br>L-Serine	D1<br>Tween 80	D2<br>D-Mannitol	D3<br>4-Hydroxy Benzoic Acid	D4<br>L-Serine	D1<br>Tween 80	D2<br>D-Mannitol	D3<br>4-Hydroxy Benzoic Acid	D4<br>L-Serine
E1<br>a-Cyclodextrin	E2<br>N-Acetyl-D-Glucosamine	E3<br>y-Hydroxybutyric Acid	E4<br>L-Threonine	E1<br>a-Cyclodextrin	E2<br>N-Acetyl-D-Glucosamine	E3<br>y-Hydroxybutyric Acid	E4<br>L-Threonine	E1<br>a-Cyclodextrin	E2<br>N-Acetyl-D-Glucosamine	E3<br>y-Hydroxybutyric Acid	E4<br>L-Threonine
F1<br>Glycogen	F2<br>D-Glucosaminic Acid	F3<br>Itaconic Acid	F4<br>Glycyl-L-Glutamic Acid	F1<br>Glycogen	F2<br>D-Glucosaminic Acid	F3<br>Itaconic Acid	F4<br>Glycyl-L-Glutamic Acid	F1<br>Glycogen	F2<br>D-Glucosaminic Acid	F3<br>Itaconic Acid	F4<br>Glycyl-L-Glutamic Acid
G1<br>D-Cellobiose	G2<br>Glucose-1-Phosphate	G3<br>a-Ketobutyric Acid	G4<br>Phenylethyl-amine	G1<br>D-Cellobiose	G2<br>Glucose-1-Phosphate	G3<br>a-Ketobutyric Acid	G4<br>Phenylethyl-amine	G1<br>D-Cellobiose	G2<br>Glucose-1-Phosphate	G3<br>a-Ketobutyric Acid	G4<br>Phenylethyl-amine
H1<br>a-D-Lactose	H2<br>D,L-a-Glycerol	H3<br>D-Malic Acid	H4<br>Putrescine	H1<br>a-D-Lactose	H2<br>D,L-a-Glycerol	H3<br>D-Malic Acid	H4<br>Putrescine	H1<br>a-D-Lactose	H2<br>D,L-a-Glycerol	H3<br>D-Malic Acid	H4<br>Putrescine

Table S2 Amount of C and rates of increase for aggregate sizes by treatments (microbial fertilizer) and controls.

Treatment	Macro-aggregate (>2,000 μm)		Macro-aggregate (250–2,000 μm)		Micro-aggregate (53–250 μm)		Silt + clay fraction (<53 μm)	
	C amount (g C kg^{-1} soil)	Increase rate (%)	C amount (g C kg^{-1} soil)	Increase rate (%)	C amount (g C kg^{-1} soil)	Increase rate (%)	C amount (g C kg^{-1} soil)	Increase rate (%)
MF1	1.91 ± 0.20Aa	20.76	1.28 ± 0.20Ad	20.01	2.10 ± 0.42Ab	15.05	1.50 ± 0.12Ac	-12.85
CK1	1.58 ± 0.14Bb	—	1.07 ± 0.01Bc	—	1.82 ± 0.02Ba	—	1.72 ± 0.01Ab	—
MF2	1.76 ± 0.06Aa	28.94	1.07 ± 0.01Ac	21.72	1.74 ± 0.21Ab	5.21	1.56 ± 0.01Aab	-16.66
CK2	1.37 ± 0.02Bb	—	0.88 ± 0.01Bc	—	1.66 ± 0.06Ab	—	1.88 ± 0.05Aa	—
MF3	1.09 ± 0.07Ab	34.00	0.72 ± 0.06Ac	25.66	1.76 ± 0.66Aa	16.4	1.59 ± 0.04Aa	-10.55
CK3	0.81 ± 0.02Bc	—	0.57 ± 0.01Bd	—	1.51 ± 0.11Bb	—	1.78 ± 0.03Aa	—
MF4	0.83 ± 0.01Ac	12.38	0.49 ± 0.01Ad	34.19	1.49 ± 0.12Ab	19.64	2.17 ± 0.37Aa	14.09
CK4	0.73 ± 0.02Ac	—	0.37 ± 0.01Bd	—	1.25 ± 0.02Bb	—	1.90 ± 0.04Aa	—
MF5	0.28 ± 0.01Ac	41.57	0.29 ± 0.01Ac	71.64	1.19 ± 0.35Ab	44.35	1.67 ± 0.25Aa	2.31
CK5	0.20 ± 0.02Ac	—	0.17 ± 0.02Bc	—	0.83 ± 0.12Ab	—	1.63 ± 0.19Aa	—

Values are means (n = 3) with standard error. Different letters within the same column indicate significant differences between treatments and controls for the same salt content at $P < 0.05$.

Table S3 Diversity indices of microbial C source utilization as affected by soil salinity and organic amendment

Soil salinity (ds/m)	Fertilizer	Diversity index		
		H`(Shannon)	E (Shannon)	D (Simpson)
0.30	No organic amendment	2.60 a	0.80 a	2.60 a
0.62		2.45 ab	0.77 ab	2.45 a
1.13		2.35 bc	0.73 ab	2.35 ab
1.45		2.06 d	0.70 ab	2.06 b
2.04		1.70 c	0.63 b	1.70 c
0.30	Organic amendment	2.35 b	0.77 ab	2.35 b
0.62		2.23 c	0.77 ab	2.17 b
1.13		2.16 c	0.69 ab	2.06 c
1.45		1.74 de	0.70 ab	1.83 c
2.04		1.65 e	0.63 b	1.41 c
Two-way ANOVA (significance)				
salinity (S)	***	***	***	***
Fertilization (F)	***	ns	ns	*
Interaction (S ×F)	***	***	*	***

Different letters within the same column indicate significant differences at $P < 0.05$ level among the ten treatments.
*** $P < 0.001$.
** $P < 0.01$.
* $P < 0.05$.
ns, $P \geq 0.05$.

Table S4 Effects of salinity and organic amendment on total soil CO_2 emissions during incubation

	Salt concentration	Organic amendment	Salt×organic amendment
df	4	1	9
F value	493.858	1759.751	2121.709
P value	<0.001	<0.001	<0.001

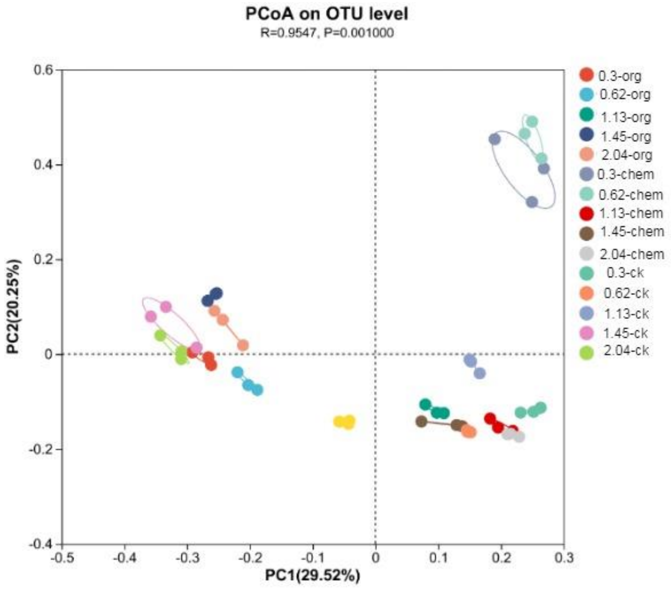

Fig S1. Principal component analysis (PCA) based on the bacterial community.

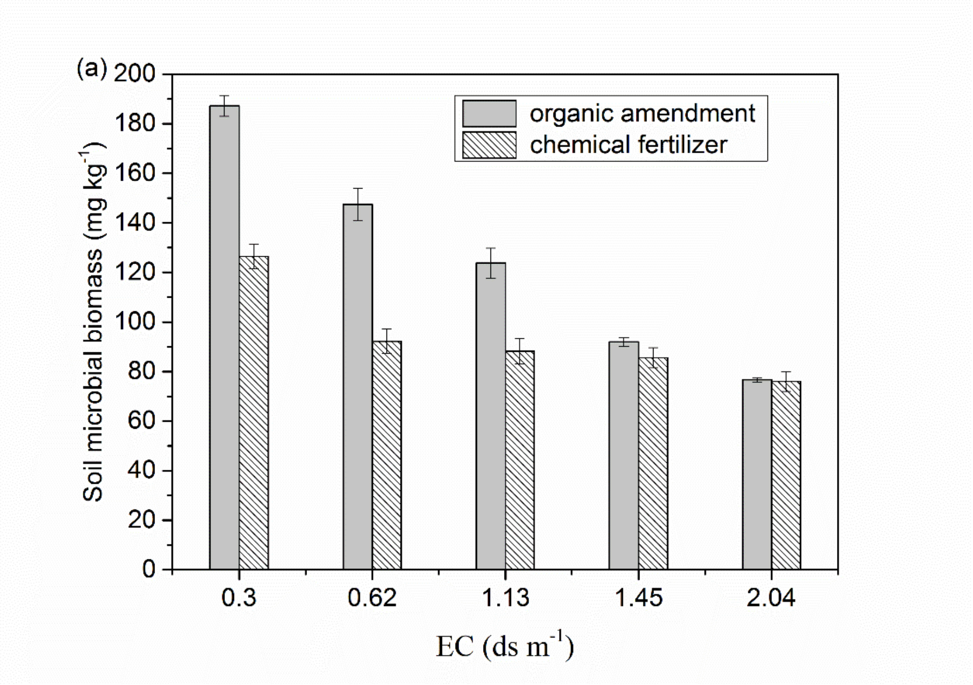

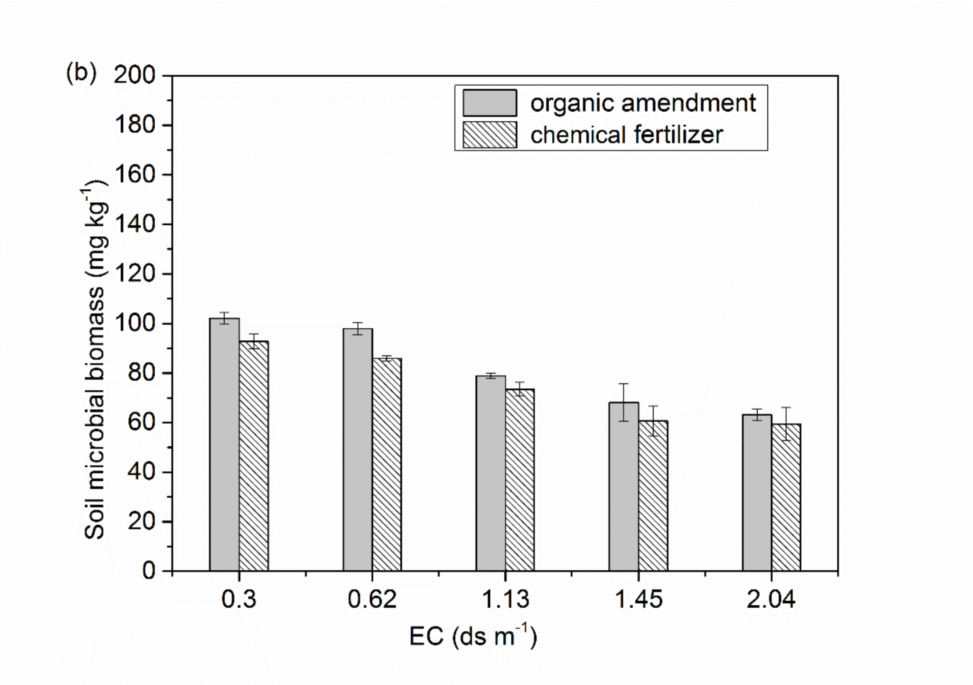

Fig. S2 Soil microbial biomass (MBC) for different salinity levels (a) MBC of soil samples at the early stage after organic amendment and (b) MBC of soil samples four months after organic amendment

Additional Experiment: R_H response to the application of organic amendment

The incubation experiment was carried out from May 10, 2021 to September 1, 2021. The soils were the same as those in the pot experiment and corresponded to five levels of EC: 0.30, 0.62, 1.13, 1.45, and 2.04 ds m^{-1}. The dried soils were first adjusted to 60% of maximal WHC. Next, 50 g of a fresh soil was placed in a 250 mL glass bottle, and the soil was pre-incubated for 7 days to stabilize the microbial activity. Then, 0.78 g of organic amendment (300 kg N ha^{-1}) was added to soil samples which were application of organic amendment treatments, and 200 mg of chemical fertilizer was added to the soil samples which were controls, to balance nutrients content. Each treatment was performed in triplicate. The soil samples were incubated in the dark for

1 days at 21 ± 1°C. During this incubation, we kept the soil at 60% of WHC by adding distilled water.

Soil respiration measurement

The measurement was based on the procedure described by Ma et al. (2016). These serum vials were removed in 2500 ml sealable tanks. Twenty milliliters of 1 mol L^{-1} NaOH was placed in every tank. The released CO_2 was absorbed by NaOH. We took the NaOH solution out after 1, 3, 6, 10 or 20 h or 1, 3, 5, 7, 10, 13, 25, 35, 65, or 95 d. The amount of CO_2 for each treatment was determined by using the titration method.

Statistical analysis

The data were analyzed using the SPSS 21 statistical software by two-way analysis of variance (ANOVA) at a significance level of 0.05. The concentrations of soil salt and organic amendment status were the independent variables. Treatment means were compared by Duncan's multiple-comparison test at $P = 0.05$. If the P value was lower, we concluded that the difference was statistically significant.

Results

Soil CO_2 emissions

A flush of CO_2 carbon (CO_2-C) emissions was analyzed just after applying organic amendment to the saline soil. For the organic amendment treatments, the initial CO_2-C was 3179.55 μg $kg^{-1}h^{-1}$ (Fig. S3b) at the EC of 0.30 ds m^{-1} in soil and decreased with

the increasing salinity. During the following 5 days, the CO_2-C emission rates in the organic amendment soils dropped sharply and decreased to 21.96–34.44% of the initial values. For the groups without application of organic amendment, the CO_2-C emissions were small and changed little; they decreased with the increasing salinity, but this decrease range was smaller than that in soil samples with organic amendment (Fig. S3a). After 35 days of incubation, the CO_2-C emission rates of the samples with organic amendment were steady and 40.51–85.16% higher as compared to samples without organic amendment. There was a significant effect of the interaction between organic amendment status and salinity on soil CO_2-C emissions (Table S4).

For the organic amendment treatments, cumulative CO_2-C emissions were also higher than in the samples without organic amendment and of the same order magnitude as the average CO_2-C emission rates at the five levels of the salinity gradient (Fig. S3c, S3d). The average cumulative CO_2-C emissions of the soil amended with organic amendment increased by 43.60%, 46.35%, 47.36%, 61.66%, and 59.85% in the order of salinity from low to high, as compared to the samples without organic amendment. During the first 5 days of incubation, for the soil samples with organic amendment, the CO_2-C emission accounted for only 15.23–20.73% of the cumulative CO_2-C emissions.

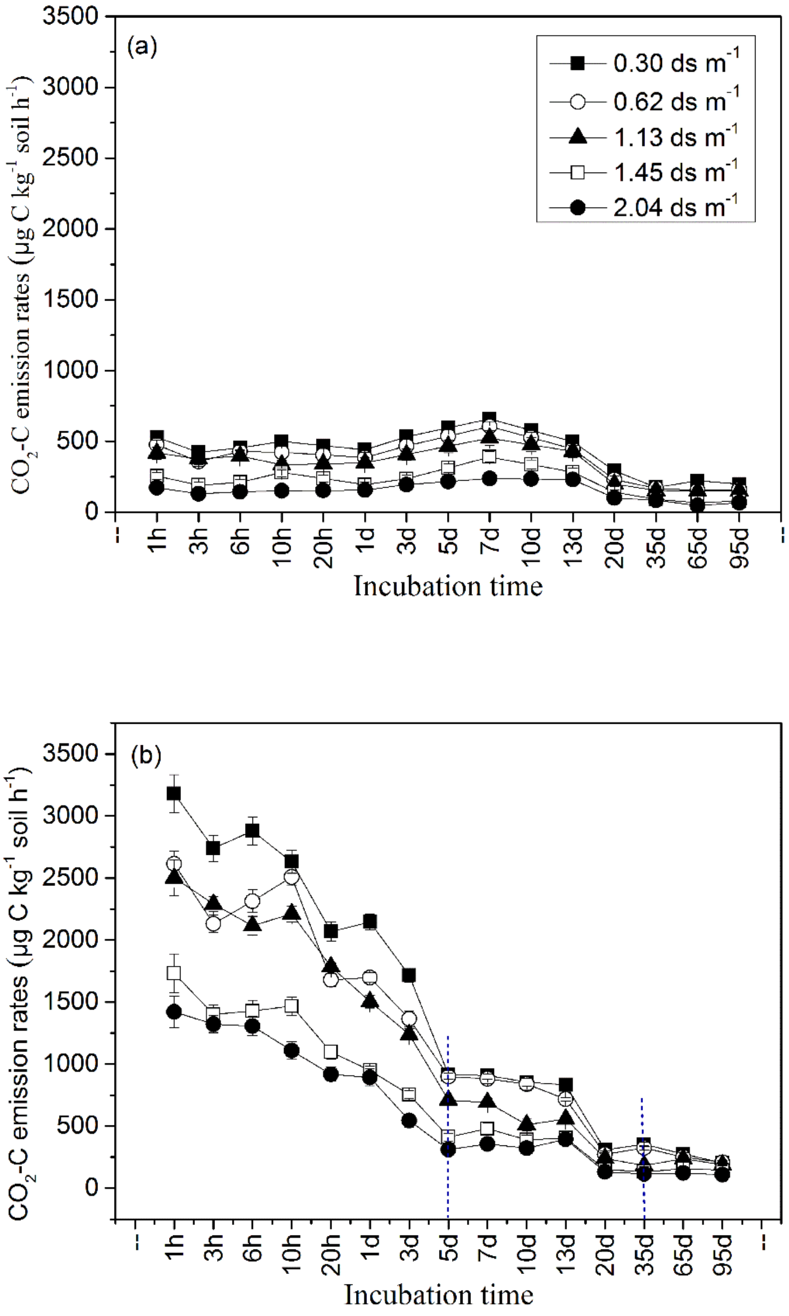

(a)
0.30 ds m-1
0.62 ds m-1
1.13 ds m-1
1.45 ds m-1
2.04 ds m-1
CO2-C emission rates (µg C kg-1 soil h-1)
Incubation time
1h
3h
6h
10h
20h
1d
3d
5d
7d
10d
13d
20d
35d
65d
95d
(b)

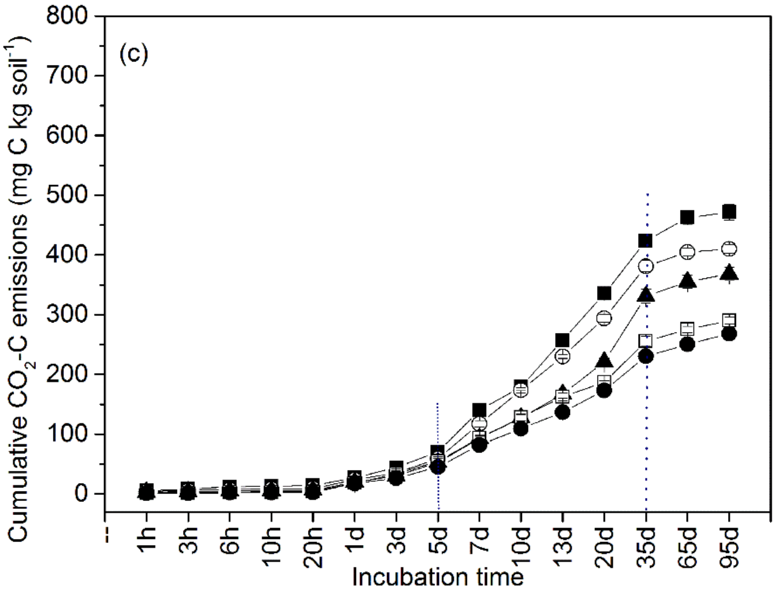

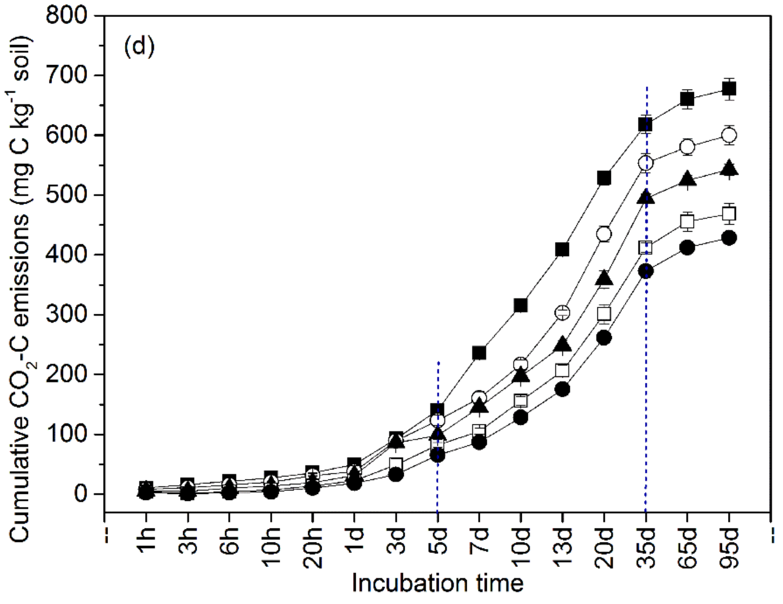

Fig. S3 Effects of organic amendment on (a) CO_2-C emission rates of soil samples without organic amendment, (b) CO_2-C emission rates of soil samples with organic amendment, (c) cumulative CO_2-C emissions of soil samples without organic amendment, and (d) cumulative CO_2-C emission rates of soil samples with organic amendment. EC = 0.30 ds m^{-1} indicates that the electric conductivity of soil is 0.30 ds

m^{-1} (■), EC=0.62 ds m^{-1} indicates the soil electric conductivity of 0.62 ds m^{-1} (◇), EC=1.13 ds m^{-1} denotes the soil electric conductivity of 1.13 ds m^{-1} (▲), EC=1.45 ds m^{-1} represents the soil electric conductivity of 1.45 ds m^{-1} (○), EC=2.04 ds m^{-1} indicates the soil electric conductivity of 2.04 ds m^{-1} (●). Values represent mean ± SE (n = 3).
